# Morphological disparity and structural performance of the dromaeosaurid skull informs ecology and evolutionary history

**DOI:** 10.1186/s12862-024-02222-5

**Published:** 2024-04-16

**Authors:** Yuen Ting Tse, Case Vincent Miller, Michael Pittman

**Affiliations:** 1 School of Life Sciences, The Chinese University of Hong Kong, Shatin, Hong Kong SAR, China; 2 Department of Earth Sciences, The University of Hong Kong, Pokfulam, Hong Kong SAR, China

**Keywords:** dromaeosaurid, morphological disparity, structural performance, skull, theropod ecology and evolutionary history

## Abstract

Non-avialan theropod dinosaurs had diverse ecologies and varied skull morphologies. Previous studies of theropod cranial morphology mostly focused on higher-level taxa or characteristics associated with herbivory. To better understand morphological disparity and function within carnivorous theropod families, here we focus on the Dromaeosauridae, ‘raptors’ traditionally seen as agile carnivorous hunters.

We applied 2D geometric morphometrics to quantify skull shape, performed mechanical advantage analysis to assess the efficiency of bite force transfer, and performed finite element analysis to examine strain distribution in the skull during biting. We find that dromaeosaurid skull morphology was less disparate than most non-avialan theropod groups. Their skulls show a continuum of form between those that are tall and short and those that are flat and long. We hypothesise that this narrower morphological disparity indicates developmental constraint on skull shape, as observed in some mammalian families. Mechanical advantage indicates that *Dromaeosaurus albertensis* and *Deinonychus antirrhopus* were adapted for relatively high bite forces, while *Halszkaraptor escuilliei* was adapted for high bite speed, and other dromaeosaurids for intermediate bite forces and speeds. Finite element analysis indicates regions of high strain are consistent within dromaeosaurid families but differ between them. Average strain levels do not follow any phylogenetic pattern, possibly due to ecological convergence between distantly-related taxa.

Combining our new morphofunctional data with a re-evaluation of previous evidence, we find piscivorous reconstructions of *Halszkaraptor escuilliei* to be unlikely, and instead suggest an invertivorous diet and possible adaptations for feeding in murky water or other low-visibility conditions. We support *Deinonychus antirrhopus* as being adapted for taking large vertebrate prey, but we find that its skull is relatively less resistant to bite forces than other dromaeosaurids. Given the recovery of high bite force resistance for *Velociraptor mongoliensis*, which is believed to have regularly engaged in scavenging behaviour, we suggest that higher bite force resistance in a dromaeosaurid taxon may reflect a greater reliance on scavenging rather than fresh kills.

Comparisons to the troodontid *Gobivenator mongoliensis* suggest that a gracile rostrum like that of *Velociraptor mongoliensis* is ancestral to their closest common ancestor (Deinonychosauria) and the robust rostra of *Dromaeosaurus albertensis* and *Deinonychus antirrhopus* are a derived condition. *Gobivenator mongoliensis* also displays a higher jaw mechanical advantage and lower resistance to bite force than the examined dromaeosaurids, but given the hypothesised ecological divergence of troodontids from dromaeosaurids it is unclear which group, if either, represents the ancestral condition. Future work extending sampling to troodontids would therefore be invaluable and provide much needed context to the origin of skull form and function in early birds. This study illustrates how skull shape and functional metrics can discern non-avialan theropod ecology at lower taxonomic levels and identify variants of carnivorous feeding.

**Supplementary Information:**

The online version contains supplementary material available at 10.1186/s12862-024-02222-5.

## Introduction

Non-avialan theropod dinosaurs were ecologically diverse [1, 2] and showed great variety in skull morphology [3–5], body size [6] and body plan [7]. However Dromaeosauridae, the quintessential “raptors”, have traditionally been painted with a broad brush as medium-sized, swift-moving macropredators [8, 9]. Recently, some key specimens [10, 11] have revealed that the characteristic ecological diversity of Theropoda is also reflected within Dromaeosauridae, but this has yet to be investigated quantitatively and with respect to their evolutionary history.

Skulls are an important morphological unit in all animals, particularly in theropods. Multiple theropod lineages saw a reduction in the forelimbs with the skull acting as the major tool for environmental manipulation [12]. Their skulls are generally more morphologically diverse than any other region of the body [13]. The most straightforward use of the theropod skull is feeding, where it is used to disassemble and often kill prey items. As such, previous studies of theropod skull shape have focused on its relationship with dietary ecology. Most of these focused on large-scale differences associated with higher-level theropod phylogeny [3–5], and ultimately were unable to demonstrate a strong relationship between shape and feeding ecology. However, there have been suggestions that cranial morphology may correlate better with ecology at lower taxonomic levels [3, 4]. This suggestion has been tested and supported to some extent in mammals, but not in theropods. For instance, correlations between skull shape and feeding are weak in carnivorans [14] but much stronger in pinnipeds [15]. Dromaeosauridae, as a well-resolved and highly-studied clade within Theropoda [16], is an ideal clade to test this hypothesis in theropod dinosaurs.

In the context of skull shape, functional metrics provide key information that help to understand ecology. Several non-avialan theropod studies have had success tracking ecological changes at large scales, though these studies are typically investigating transitions to herbivory [17–20] rather than the carnivorous niches as expected for dromaeosaurids e.g. [21–23]. Dromaeosaurids, once again, serve as an ideal group to further explore how carnivorous non-avialan theropods differed functionally. Dromaeosaurids purportedly had a broad range of cranial morphology [10, 11, 24]. Different species also cohabitated in palaeoecosystems with diverse climates and environments [22, 25–27], implying diverse niches to limit competition with one another.

Here, we investigate the cranial shape and bite mechanics of Dromaeosauridae (Table 1) in a phylogenetic context to investigate potential ecological differences in the clade and the possible evolutionary pathways they took. We capture size and shape data with geomorphic morphometrics (GM), and bite performance with both mechanical advantage (MA) and finite element analysis (FEA). Previous non-avialan theropod studies had only applied up to two of these techniques; by combining all three techniques here for the first time, we can create a more complete picture of the form-function dynamics at play. Combining these results with phylogenetic comparative methods (PCMs) allows us to craft a more complete picture of dromaeosaurid ecological diversity and its development through time.

**Table 1:** All specimens used in this study. Table showing all specimens which have been included in the current study, with genus, species, and clade labelled. The specimen number and sources of the specimen photos are also listed. Institutional abbreviation: AMNH, American Museum of Natural History; BMNHC, Beijing Museum of Natural History; IGM, Institute of Geology, Mongolian Academy of Sciences; IVPP, Institute for Vertebrate Paleontology and Paleoanthropology; MPC, dinosaur collection of the Paleontological and Geological Center, Mongolian Academy of Sciences; UALVP, University of Alberta, Laboratory of Vertebrate Paleontology; YPM, Yale Peabody Museum, Yale University

Genus	Species	Clade	Specimen number	Citation, Figure number from citation
*Deinonychus*	*antirrhopus*	Velociraptorine	YPM 5210, YPM 5230	[28], Figure 4
*Dromaeosaurus*	*albertensis*	Dromaeosaurinae	AMNH 5356	[29], Figure 1
*Halszkaraptor*	*escuilliei*	Non-eudromaeosaurian dromaeosaurid / “Halszkaraptorinae”	MPC D-102/109	[10], Figure 2
*Linheraptor*	*exquisitus*	Velociraptorinae	IVPP V16923	[30], Figure 1
*Microraptor*	*zhaoianus*	Microraptorinae	BMNHC PH881	[31], Figure 2
*Saurornitholestes*	*langstoni*	Velociraptorinae	UALVP 55700	[32], Figure 1
*Sinornithosaurus*	*millenii*	Microraptorinae	IVPP uncatalogued	[24], Figure 23C
*Tsaagan mangas*	*mangas*	Velociraptorinae	IGM 100/1015	[33], Figure 3
*Velociraptor*	*mongoliensis*	Velociraptorinae	AMNH FR 6516	[33], Figure 6
*Velociraptor*	*mongoliensis*	Velociraptorinae	IGM 100/25	[36], Figure 1
*Gobivenator*	*mongoliensis*	Troodontidae	MPC D-100/86	[37], Figure 3

## Results

### Geometric morphometric principal component analyses

#### Dataset (a): least number of landmarks, largest number of taxa

**Fig. 1:**
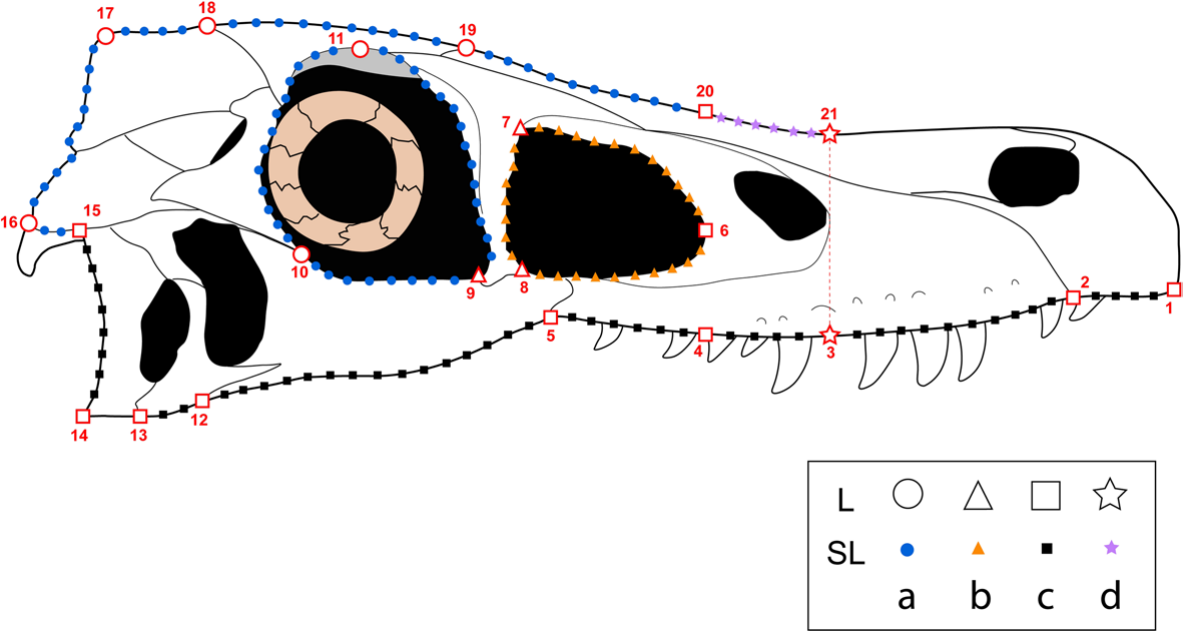
GM landmarks on a *Velociraptor mongoliensis* skull. Line drawing of a *Velociraptor mongoliensis* skull (based on IGM 100/25) with the maximum number of traditional landmarks and semi-landmarks labelled, as in dataset (d). Circles indicate landmarks in all datasets, triangles those added in dataset (b), squares those added in dataset (c), and stars those added in dataset (d)

**Fig. 2:**
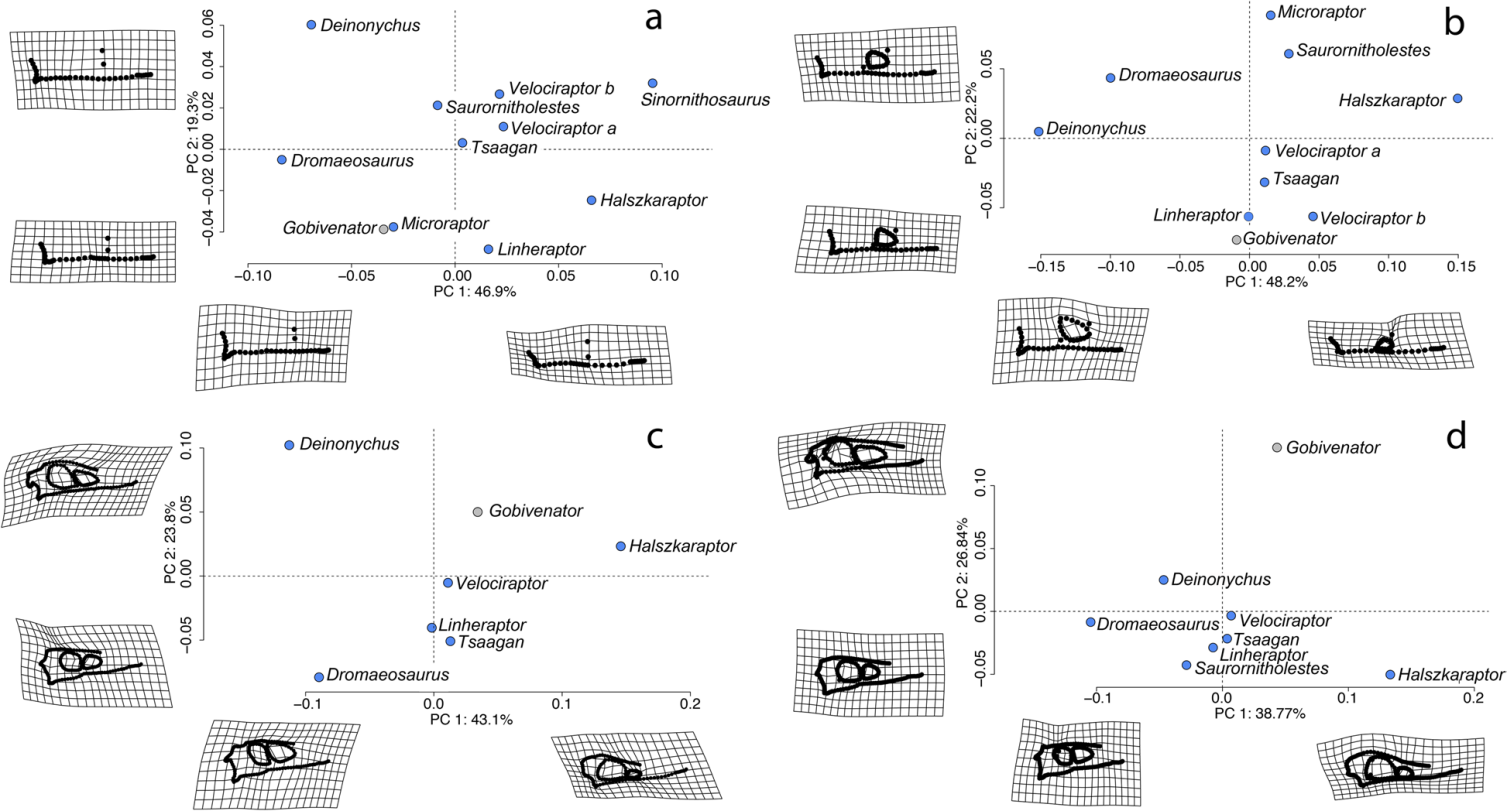
Results of principle component analyses for all four datasets. PCA graphs based on GM data. Deformation grids for positive and negative ends of each PC are placed next to the corresponding axes. a: dataset (a), least number of landmarks; b: dataset (b), intermediate number of landmarks; c: dataset (c), largest number of landmarks; d: dataset (d), reconstruction dataset with *Sinornithosaurus millenii* excluded. Abbreviations: *Velociraptor* a, specimen AMNH FR 6516; *Velociraptor* b, specimen IGM 100/25

This dataset uses 52 landmarks examining 10 species (Fig. 1, Table 2). PC1 and PC2 explain 66.2% of the cranial morphological variations observed among the 11 specimens (PC1: 46.9%; PC2: 19.3%) (Fig. 2). Positive PC1 scores describe an anteroposteriorly elongated skull and snout, dorsoventrally short and posteriorly deflected quadrate, and a posterior translation of the anterior border of the antorbital fenestra. Positive PC2 scores describe an anteroposteriorly elongate maxilla and a dorsal translation of the anterior border of the antorbital fenestra. This dataset has significant phylogenetic signal in PC1 and the shape data overall (Table 3).

**Table 2:** Organization of the four subsets of data. Table showing the species, number of traditional landmarks, number of semi-landmarks, and total number of landmarks in datasets (a) to (d). For dataset (d), two sets of data have been collected. One set has included *Sinornithosaurus millenii* and one set has excluded it as its published reconstruction is suspect (see Geometric Morphometrics Methods for details)

Dataset	Species included	Number of traditional landmarks	Number of semi-landmarks
Dataset (a): Least number of landmarks	*Deinonychus antirrhopus* *Dromaeosaurus albertensis* *escuilliei* *Linheraptor exquisitus* *Microraptor zhaoianus* *Saurornitholestes langstoni* *Sinornithosaurus millenii* *Tsaagan mangas* *Velociraptor mongoliensis* × 2 *Gobivenator mongoliensis*	10	42 (Six curves of seven semi-landmarks)
Dataset (b): Intermediate number of landmarks	*Deinonychus antirrhopus* *Dromaeosaurus albertensis* *Halszkaraptor escuilliei* *Linheraptor exquisitus* *Microraptor zhaoianus* *Saurornitholestes langstoni* *Tsaagan mangas* *Velociraptor mongoliensis* × 2 *Gobivenator mongoliensis*	13	63 (9 curves of seven semi-landmarks)
Dataset (c): Largest number of landmarks	*Deinonychus antirrhopus* *Dromaeosaurus albertensis* *Halszkaraptor escuilliei* *Linheraptor exquisitus* *Tsaagan mangas* *Velociraptor mongoliensis* (IGM 100/25) *Gobivenator mongoliensis*	19	187 (17 curves of 11 semi-landmarks)
Dataset (d): Reconstruction dataset	*Deinonychus antirrhopus* *Dromaeosaurus albertensis* *Halszkaraptor escuilliei* *Linheraptor exquisitus* *Saurornitholestes langstoni* Sinornithosaurus millenii* *Tsaagan mangas* *Velociraptor mongoliensis* (IGM 100/25) *Gobivenator mongoliensis*	21	209 (19 curves of 11 semi-landmarks)

**Table 3:** Phylogenetic signal of PCA data. K_mult_ for whole shape data and Blomberg’s K for individual shape PCs are provided. Parameters with *p*-values significant at the *p* < 0.05 level are bolded. Only PC1, PC2, and significant PC(s) are shown in the table for brevity. For complete data, see supplementary information

**Dataset**	**K**_**mult**_	***p*** **-value**	
a	0.0098	0.012	
b	0.0108	0.015	
c	0.691	0.044	
d (excluding *S. millenii*)	0.812	0.005	
	**PC**	**K**	***p*** **-value**
a	***PC1***	***0.41***	***0.016***
	PC2	0.0212	0.194
	***PC6***	***0.415***	***0.025***
	***PC9***	***0.264***	***0.028***
b	PC1	0.0208	0.395
	PC2	0.0234	0.197
c	PC1	0.863	0.143
	PC2	0.686	0.459
	***PC3***	***1.08***	***0.038***
d (excluding *S. millenii*)	***PC1***	***1.3***	***0.007***
	PC2	1.29	0.083
**Dataset**	**K**_**mult**_	***p*** **-value**	
a	***0.0098***	***0.012***	
b	***0.0108***	***0.015***	
c	***0.691***	***0.044***	
d (excluding *S. millenii*)	***0.812***	***0.005***	
	**PC**	**K**	***p*** **-value**
a	***PC1***	***0.41***	***0.016***
	PC2	0.0212	0.194
	***PC6***	***0.415***	***0.025***
	***PC9***	***0.264***	***0.028***
b	PC1	0.0208	0.395
	PC2	0.0234	0.197
c	PC1	0.863	0.143
	PC2	0.686	0.459
	***PC3***	***1.08***	***0.038***
d (excluding *S. millenii*)	***PC1***	***1.3***	***0.007***
	PC2	1.29	0.083

**Fig. 3:**
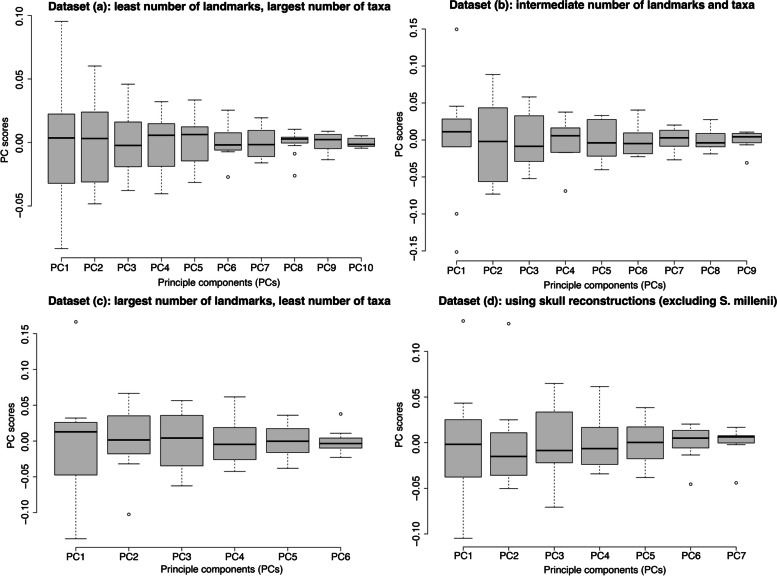
Boxplots of principal components. Boxplots generated from outlier tests of all four datasets. X-axis represents each of the principal components found in the datasets. Y-axis represents the PC scores. The black line in each of the box represents the median PC score of the individual PCs. The small circles on the graph represents the outliers in each PC and thus identify taxa with unusual skull shape

In outlier tests, outliers were identified in PC6 and PC8 (Fig. 3). For PC6, one outlier was identified: *Microraptor* is a lower outlier. Negative PC6 scores describe an anteroposteriorly less elongated premaxilla, a dorsoventrally flatter maxilla, and a dorsoventrally straight quadrate (Fig. S1). For PC8, two outliers were identified: *Velociraptor mongoliensis* (specimen IGM 100/25) and *Dromaeosaurus albertensis* are both lower outliers. Negative PC8 scores describe a more curved ventral edge of skull and dorsoventrally straight quadrate (Fig. S1). Although no outlier has been identified, the standard deviation for PC1 is much greater than other PCs (Fig. 3).

#### Dataset (b): intermediate number of landmarks and taxa

This dataset uses 76 landmarks examining 9 species (Fig. 1, Table 2). PC1 and PC2 explain 70.4% of the cranial morphological variations observed among the ten specimens (PC1: 48.2%; PC2: 22.2%) (Fig. 2). Positive PC1 scores describe an anteroposteriorly elongate and dorsoventrally short skull; anteroposteriorly elongate snout, jugal, and area posterior to antorbital fenestrae; posterior-deflected of the dorsal end of quadrate; and anteroposteriorly and dorsoventrally short antorbital fenestra. Positive PC2 scores describe a dorsoventrally short and posteriorly deflected quadrate, and an anteroposteriorly elongated snout and area posterior to antorbital fenestrae. This dataset has significant phylogenetic signal in PC4 and the shape data overall (Table 3).

In outlier tests, outliers were identified in PC1, PC4, and PC9 (Fig. 3). For PC1, three outliers were identified: *Halszkaraptor escuilliei* is an upper outlier, and *Dromaeosaurus albertensis* and *Deinonychus antirrhopus* are lower outliers. For PC4, one outlier was identified: *Gobivenator mongoliensis* is a lower outlier. Negative PC4 scores describe an anteroposteriorly expanded premaxilla and jugal, and a slightly anteroposteriorly expanded antorbital fenestra (Fig. S1). For PC9, one outlier was identified: the *Velociraptor mongoliensis* specimen IGM 100/25 is the lower outlier. Negative PC9 scores describe an anteroposteriorly more expanded premaxilla and jugal, a dorsoventrally straight quadrate, and a more circular antorbital fenestrae (Fig. S1).

#### Dataset (c): largest number of landmarks, least number of taxa

This dataset uses 206 landmarks examining 7 species (Fig. 1, Table 2). Together, PC1 and PC2 explain 66.9% of the cranial morphological variations observed among the six specimens (PC1: 43.1%; PC2: 23.8%) (Fig. 2). Positive PC1 scores describe an anteroposteriorly slightly elongated skull and snout, dorsoventrally compressed snout and frontal, anteroposteriorly elongated jugal, dorsoventrally short quadrate, rounded dorsal and posterior edges of the cranium, antorbital fenestra anteroposteriorly and dorsoventrally short, orbit being circular (in contrast to being elliptical), and smaller postorbital space. Positive PC2 scores describe a dorsoventrally compressed snout (less extreme than in PC1), anteroposteriorly elongate skull roof, a dorsoventrally concaved skull roof region, a dorsoventrally posteriorly concaved quadrate, and an angular antorbital fenestra. This dataset has significant phylogenetic signal in PC3 and the shape data overall (Table 3).

In outlier tests, three outliers were recovered. *Halszkaraptor escuilliei* is an upper outlier of PC1, *Dromaeosaurus albertensis* is a lower outlier of PC2, and *Velociraptor mongoliensis* is an upper outlier of PC6 (Fig. 3). Positive PC6 scores describe an anteroposteriorly compressed premaxilla and expanded maxilla, dorsoventally more dome-shaped parietal, a more circular orbit, and more posteriorly expanded squamosal (Fig. S1).

#### Dataset (d): using skull reconstructions (excluding Sinornithosaurus millenii)

This dataset uses 230 landmarks examining 8 species (Fig. 1, Table 2). Together, PC1 and PC2 explain 65.6% of the cranial morphological variations observed among the seven specimens (PC1: 38.8%; PC2: 26.8%) (Fig. 2). Positive PC1 scores describe an anteroposteriorly more elongated skull, dorsoventrally compressed and anteroposteriorly elongate snout, anteroposteriorly elongated skull and jugal, dorsoventrally short and posteriorly deflected quadrate, rounded posterior end of skull, reduced antorbital fenestra, a dorsoventrally and anteroposteriorly expanded and circular orbit, and a smaller postorbital area. Positive PC2 scores describe a dorsoventrally concave skull roof region, a posteriorly protruded squamosal, a anteroposteriorly expanded frontal region, and an anteroposteriorly expanded and angular antorbital fenestra. This dataset has significant phylogenetic signal in PC1, PC2, and the shape data overall (Table 3).

Outliers were identified along PC1, PC2, PC6, and PC7 (Fig. 3). For PC1, one outlier was identified: *Halszkaraptor escuilliei* is an upper outlier. For PC2, one outlier was identified: *Gobivenator mongoliensis* is an upper outlier. For PC6, one outlier was identified: *Tsaagan mangas* is a lower outlier. Positive PC6 scores describe a dorsoventrally dome shaped skull roof, a dorsoventrally straight quadrate, and a more circular orbit (Fig. S1). For PC7, one outlier was identified: *Velociraptor mongoliensis* (specimen IGM 100/25) is an upper outlier. Positive PC7 scores describe a less posteriorly protruded squamosal and a slightly dorsoventrally expanded posterior skull and snout (Fig. S1).

### Centroid size comparisons

**Fig. 4:**
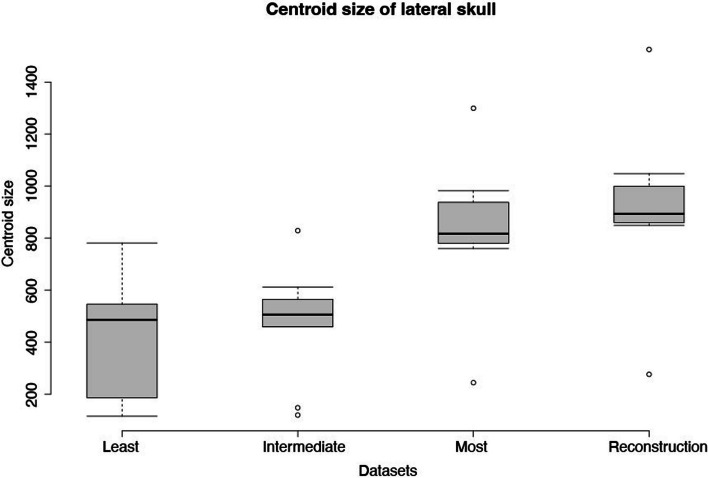
Boxplot of centroid size for all four datasets. X-axis represent the four datasets; "least", "intermediate", and "most" refer to the number of landmarks in the dataset. Y-axis represents the centroid size of the specimens. The black line in each of the box represents the median centroid size of the dataset. The small circles on the graph represents outliers in the datasets and thus identify taxa with unusual skull size. This visualisation serves chiefly to illustrate outlier taxa easily; centroid sizes cannot be compared between datasets due to centroid size increasing with an increasing number of landmarks

Average centroid size for a dataset increases with the number of landmarks (Fig. 4). As the number of landmarks included in the dataset increases, more distances between the centroid and the landmarks are added together, making the centroid size necessarily larger. Thus, centroid sizes are only comparable within datasets, not between them. Outlier tests were performed for all datasets. Three datasets have outliers identified (Fig. 4). *Deinonychus antirrhopus* is an upper outlier and *Halszkaraptor escuilliei* is a lower outlier in dataset (c). *Deinonychus antirrhopus* is an upper outlier and *Halszkaraptor escuilliei* is a lower outlier in datasets (b) and (d). *Microraptor* is also a lower outlier in dataset (b).

### Mechanical advantage (MA)

When using temporal group to determine the in-lever, *Dromaeosaurus albertensis* shows the highest MA following by *Deinonychus antirrhopus* (Table 4). Both species have MA above 0.3 and only differ from each other for 0.004. *Halszkaraptor escuilliei* has the smallest MA among all species and the value is below 0.2. Most of the species included have MA above 0.2 but below 0.3. When using the quadrate muscle group to determine the in-lever, *Gobivenator mongoliensis* (MA = 0.488) shows the highest MA following by *Linheraptor exquisitus* (MA = 0.478) and *Halszkaraptor escuilliei* (MA = 0.461). The majority of the species have quadrate group [sensu 28] based MA above 0.4. The only two species with MA lower than this is *Velociraptor mongoliensis* (MA = 0.395) and *Microraptor* (MA = 0.334). *Velociraptor mongoliensis* has MA just below 0.4, whereas *Microraptor* has the smallest quadrate MA among all species. For differences between temporal MA and quadrate group-based MA (∆MA) of all species, *Halszkaraptor escuilliei* shows the greatest value (∆MA = 0.281) due to the high quadrate MA and very low temporal MA. *Dromaeosaurus albertensis* shows the least amount of difference (∆MA = 0.115) since both of its temporal MA and quadrate MA are relatively high.

**Table 4:** FEA and MA Results. Mesh-weighted arithmetic mean (MWAM) strain, mechanical advantage of temporal muscle group (temporal MA), mechanical advantage of quadrate muscle group (quadrate MA), and fenestrae to area ratio were calculated for all species with FEA models. Highest and lowest values are bolded in each column. MWAM strain and fenestrae-surface area could not be calculated for *Microraptor zhaoianus* due to poor preservation. However, since the rough locations of the origins and insertion of the temporal and quadrate muscle groups could be estimated from the specimen, temporal MA and quadrate MA could be obtained for *Microraptor zhaoianus*

Taxon	MWAM strain (με)	Temporal MA	Quadrate MA	Fenestrae/surface area ratio
*Deinonychus antirrhopus*	579	0.302	0.44	0.524
*Dromaeosaurus albertensis*	399	***0.306***	0.421	0.421
*Gobivenator mongoliensis*	***645***	0.274	***0.488***	***0.631***
*Halszkaraptor escuilliei*	386	***0.18***	0.461	0.381
*Linheraptor exquisitus*	434	0.272	0.478	0.405
*Tsaagan mangas*	550	0.259	0.434	***0.345***
*Velociraptor mongoliensis*	***360***	0.242	0.395	0.485
*Microraptor zhaoianus*	–	0.214	***0.334***	–

### Finite element analysis

**Fig. 5:**
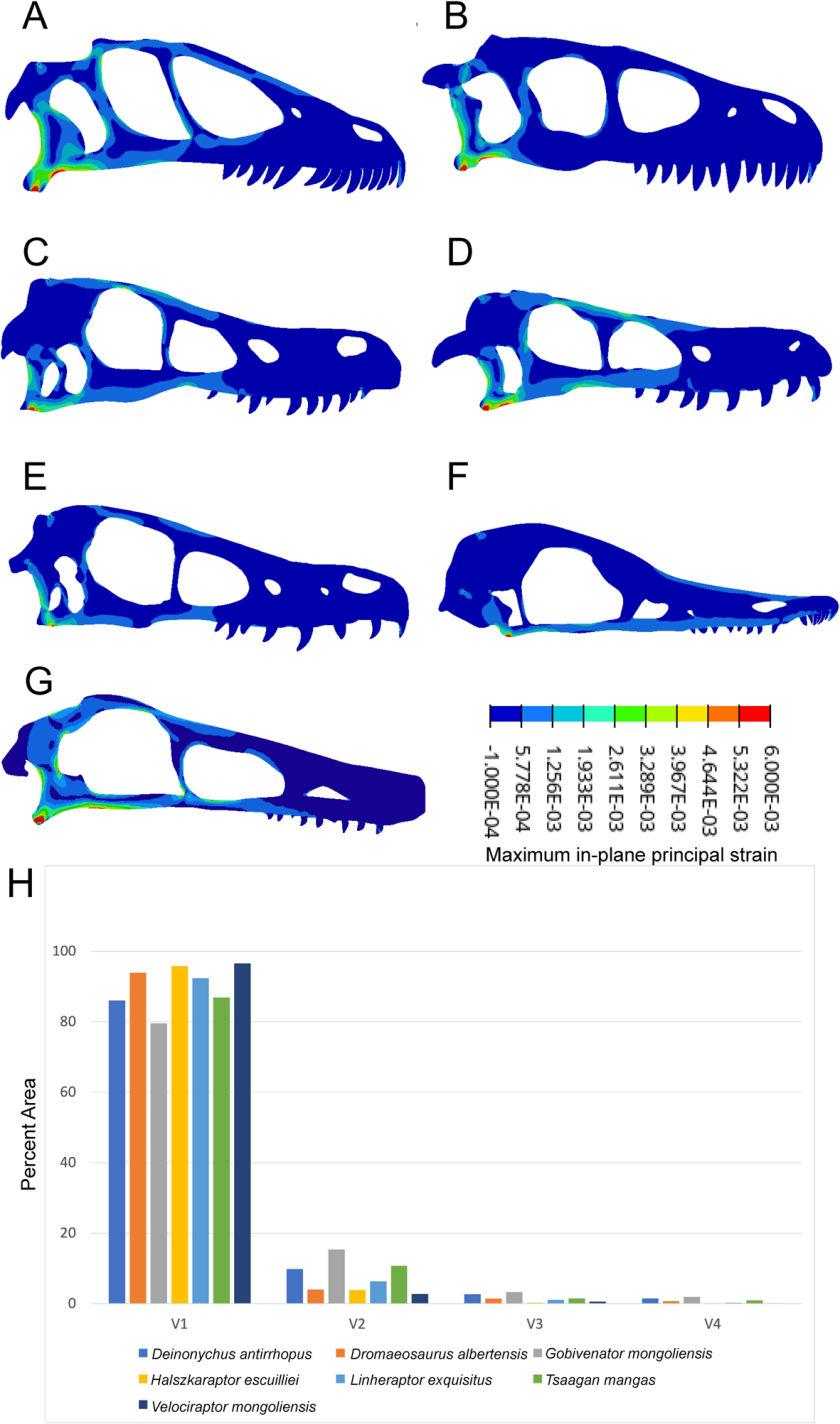
Finite element analysis results. FEA contour plots (A–F) and bar graph (G) representing results from the intervals method. Negative strain values indicate compressive strain, positive values indicate tensile strain. A: *Deinonychus antirrhopus*, B: *Dromaeosaurus albertensis*, C: *Linheraptor exquisitus*, D: *Tsaagan mangas*, E: *Velociraptor mongoliensis* (IGM 100/25), F: *Halszkaraptor escuilliei*, G: *Gobivenator mongoliensis*. H: Plot of intervals strain data. X-axis denotes intervals of strain; V1 indicates the interval of lowest strain, V4 the interval of highest strain. The y-axis indicates the percentage of the total area of each model experiencing that level of strain

In all models, the greatest strain is observed at the jaw joint (Fig. 5). Three patterns of cranial strain were identified among the models. The first pattern is observed in *Deinonychus antirrhopus* and *Dromaeosaurus albertensis*. They experience relatively high tensile strain at the posterior end of their skulls, especially in the quadrate, squamosal, parietal, and quadratojugal (Fig. 5A, B). For *Deinonychus antirrhopus*, tensile strain is also relatively high on the ventral edge of jugal and frontal, dorsal end of postorbital, and anterior and posterior edges of lacrimal. For *Dromaeosaurus albertensis*, greater proportion of the cranial surface experiences compressive strain rather than tensile strain in contrast to *Deinonychus antirrhopus*.

The second pattern is seen in *Linheraptor exquisitus*, *Tsaagan mangas*, and *Velociraptor mongoliensis* (Fig. 5C, D, E). Relatively high tensile strain is observed along the dorsal and ventral edge of the skulls, particularly in quadratojugal and jugal. *Tsaagan mangas* and *Linheraptor exquisitus* experience high tensile strain at posterior end of maxilla and dorsal edge of the skulls. The magnitude of tensile strain of *Velociraptor mongoliensis* in these areas is comparatively small, as well as at the anterior end of frontal and the posterior end of nasal. High tensile strain is also observed on the anterior dorsal edge of the antorbital fenestrae in *Tsaagan mangas*. All three specimens experience compressive strains in similar regions.

The Third pattern is seen only in *Halszkaraptor escuilliei* (Fig. 5F). The highest tensile strain is at the ventral ends of the quadrate, quadratojugal, and jugal, and the ventral end of orbit where it contacts jugal. Low compressive strain occurs at the anterior end and dorsal edge of the maxilla, lacrimal, postorbital, frontal, parietal, nuchal crest, and paraoccipital process.

The Troodontid *Gobivenator mongoliensis* experiences strain pattern that is similar to the first and second pattern (Fig. 5G). It experiences relatively high tensile strain at the posterior end of their skulls, especially in the quadrate, squamosal, parietal, quadratojugal, and dorsal and ventral edge of skull. In comparison to the dromaeosaurids studied, a greater area on the posterior end and ventral edge of the skull of *Gobivenator mongoliensis* experiences high strain.

*Gobivenator mongoliensis* experiences the highest mesh-weighted arithmetic mean (MWAM) strain, a summary statistic of an FEA model’s average strain, among all models (Table 4). It is followed by *Deinonychus antirrhopus*, *Tsaagan mangas*, *Linheraptor exquisitus*, *Dromaeosaurus albertensis*, *Halszkaraptor escuilliei*, and *Velociraptor mongoliensis*. The data gleaned from the intervals method (Fig. 5H), which shows the percent area of models under various levels of strain, show the majority of the area in each model is under relatively little strain (interval 1). *Dromaeosaurus albertensis*, *Halszkaraptor escuilliei*, and *Velociraptor mongoliensis* all have very little area in any interval beyond the first. *Linheraptor exquisitus* and *Tsaagan mangas* have a spike of strain in interval 2, but minimal area in the high strain intervals 3 and 4. *Deinonychus antirrhopus* and *Gobivenator mongoliensis* both have noticeable area within the high strain intervals, exhibiting similar patterns of reduced area with increasing interval strain.

There are no significant relationships between skull length or LOG skull length to MWAM strain (Fig. S2). The ratio of total skull fenestra area to total skull area and individual skull fenestrae area to total skull area also shows no significant relationship to MWAM strain in phylogenetic generalised least squares (PGLS) analysis (Table S1).

### Phylogenetic comparative methods

#### Phylogenetic generalized least squares (PGLS) analyses

For the PGLS regression analyses which compared shape with MA, PC1 and MA of the temporal muscle group show significant relationships in all datasets (Table 5). Increase in PC1 scores correlates with increase in MA. Overall, about 74.9 to 85.9% of the variations in MA of temporal muscle group could be explained by variations in PC1 scores. Besides PC1, none of the other PCs produced significant results when regressed against MA calculated based on temporal muscle group. For the PCs regressed quadrate muscle group, PC5 in dataset (c) and PC7 in dataset (d) show significant relationships. Both increase in PC5 scores in dataset (c) and increase in PC7 scores in dataset (d) correlates with increase in MA. PC5 explains 53.2% of the variation in MA of quadrate muscle group; similarly, PC7 explains 59.5% of the variation in MA of quadrate muscle group. PGLS regression analyses which compared centroid sizes with temporal muscle group MA all recovered significant relationships, but they show no significant correlations with the quadrate muscle group MA (Table S2). No PGLS regression analyses which compared shape with MWAM strain or centroid sizes with MWAM strain recovered significant relationships (Table S3).

**Table 5:** Phylogenetic generalized least square (PGLS) regression results. PGLS of the correlations between shape (PCs) and mechanical advantage (MA) of temporal and quadrate muscle groups. Only PCs with significant correlations with MA are included in the table. No significant correlations were found between shape and MWAM strain, centroid size and MA, and centroid size and MWAM strain

	***p***-value	R^**2**^
**Dataset (a): Least number of landmarks**		
PC1 ~ Temporal	0.00289	0.826
**Dataset (b): Intermediate number of landmarks**		
PC1 ~ Temporal	0.00167	0.859
**Dataset (c): Largest number of landmarks**		
PC1 ~ Temporal	0.00277	0.829
PC5 ~ Quadrate	0.0382	0.816
**Dataset (d): Reconstruction dataset (excluding** ***S. millenii*** **)**		
PC1 ~ Temporal	0.0074	0.749
PC7 ~ Quadrate	0.0256	0.595

## Discussion

### Geometric morphometrics

Shape variation in dromaeosaurid skulls is primarily described by variation in skull length, snout length and height, lateral temporal fenestra size, orbit shape, and antorbital fenestra size. These traits are all captured in PC1 of GM PCA (explaining 38.8 to 48.2% of the variance; Fig. 2). Previous GM studies of skulls across non-avian Theropoda, which only included a maximum of four dromaeosaurid taxa, also recovered skull length [3], snout depth and length, and lateral temporal fenestra size [3, 4] as key components of PC1 (explaining 46.2 to 56.9% of the variance in [3], and 34.4% in [4]). Orbit shape and antorbital fenestra size were recovered as less influential in these studies, as components of PC2 (explaining 19.5 to 23.1% of the variance in [3], and 17.1% in [4]). Brusatte et al. [3] also reported naris shape as a major component of PC1; poor preservation prevented us from landmarking this feature. So in general, the major spectrum of shape in dromaeosaurids is consistent with trends in theropods overall and this spectrum explains a similar amount of the overall shape variance, but the antorbital fenestra and orbit shape experiences more significant variation within this clade than the average across Theropoda.

Previous studies of skull shape across Theropoda [3, 4] recovered PC1s and PC2s defined by unrelated traits (e.g. skull or snout length in PC1 vs orbit length in PC2). In contrast, many of the shape variations describing our PC2 recapitulate those defining PC1. This leads to the question of whether dromaeosaurids may have experienced developmental constraints, in which a high level of integration is present and skull shape is restricted to a single morphological trajectory (Fig. S3) [38–40]. Such constraints have been observed in skulls of other vertebrate groups such as icefish [40] and bats [38]. The morphological trajectory can be observed among Dromaeosaurids in datasets (c) and (d). But no such trajectory is observed in datasets (a) and (b). These datasets differ both in the taxa included and the inclusion of dorsal cranium landmarks, so the difference in the apparent trajectory could be driven by taxon or landmark sampling. To test which, we excluded dorsal cranium landmarks from datasets (c) and (d) (Fig. S4). Removal of the dorsal cranial landmarks resulted in a loss of the morphological trajectory, implying that the loss of trajectory in datasets (a) and (b) results from not including these landmarks rather than the increase in sample size. This also implies the dorsal cranium is important for understanding cranial integration in dromaeosaurids as in major clades within reptiles [41]. Future investigations should examine the level of cranial integration and modularity among dromaeosaurids to better interpret this observed morphological trajectory.

To test if the trajectory observed might be caused by allometry in the datasets, we performed linear regression to investigate the correlations between PC1 and PC2 with skull centroid size (Table S4). PC1 shape values in datasets (b), (c), and (d) show significant correlations with centroid size. This suggests that morphological differences represented by PC1 is presenting a mix of the actual shape and allometric effect of the skulls. When the troodontid outgroup is included the correlation between skull shape and size is still significant, so this allometric relationship potentially extends to the whole of Deinonychosauria. An increased sample size in future studies is necessary to examine this possibility in more detail.

Differences in dromaeosaurid skull shape generally follow phylogenetic trends. *Halszkaraptor escuilliei* has most anteroposteriorly elongated skull and most anteroposteriorly elongated and dorsoventrally compressed snout in the study sample (Fig. 2C,D), and is the only included Halszkaraptorine. The same general shape appears to be maintained in the Halszkaraptorine *Natovenator polydontus* [11], but the skull is too incomplete to confirm this quantitatively. *Deinonychus antirrhopus* and *Dromaeosaurus albertensis*, both early-diverging eudromaeosaurs [42], exhibit the opposite morphology from halszkaraptorines with anteroposteriorly short skulls and dorsoventrally tall snouts (Fig. 2). We interpret this more robust skull as a derived eudromaeosaurian condition, as both the early-diverging *Saurornitholestes langstoni* and late-diverging *Velociraptor mongoliensis, Linheraptor exquisitus, and Tsaagan mangas* [42] display skull morphology intermediate between *Deinonychus antirrhopus*, *Dromaeosaurus albertensis*, and *Halszkaraptor escuilliei* (Fig. 2). The phylogenetic position of *Deinonychus antirrhopus* is notably unstable e.g. [16, 42], though, so determining whether the robust condition evolved once or multiple times is currently impossible. Using the skull shape of the troodontid *Gobivenator mongoliensis* as an outgroup only slightly clarifies this matter. The cranial region of its skull is taller than most dromaeosaurids, most similar to *Deinonychus antirrhopus*, while its rostrum is low and gracile, similar to *Velociraptor mongoliensis*. While it is possible this indicates split trajectories towards either more robust rostra or gracile crania, we suspect different evolutionary pressures on the troodontid skull skew these findings. For instance, troodontids are hypothesised to have been nocturnal and possibly more intelligent than other non-avian theropods [43] associated with larger eyes and brains and thus selection for a more expanded cranium. If we assume this hypothesis of cranium shape selection is correct, then the common ancestor of Dromaeosauridae and Troodontidae did likely have a gracile rostrum and a relatively low cranium. This would corroborate the hypothesis that *Deinonychus antirrhopus* and *Dromaeosaurus albertensis* represent a derived condition of skull shape.

Our shape data enables us to comment on some of the purported cranial adaptations for aquatic predation in *Halszkaraptor escuilliei*. In particular, we can support that its platyrostral snout is indeed a morphological anomaly among dromaeosaurids. However, we contest that this condition is indicative of aquatic predation. The source that [10] cites claiming platyrostry is indicative of piscivory [35] does not, in fact, make this claim. The relevant section describes longirostry (anteroposteriorly long and mediolaterally narrow rostrum) with a terminal rosette (ovoid mediolateral broadening of premaxilla), not platyrostry: “The jaws are long and very narrow from side to side; they are expanded horizontally at the anterior end […] this spatulate expansion forms a ‘terminal rosette’, not unlike the corresponding region of the skull of a modern gavial” [35 , p.61]. Longirostry has indeed evolved repeatedly in specialist piscivores [44, 45], but this is not the morphology observed in *Halszkaraptor escuilliei*. Hone and Holtz [46] do show that aquatic and semi-aquatic animals (irrespective of diet) tend to have dorsoventrally short skulls, but this morphological distinctiveness starts to break down below a skull length of 500 mm (their Fig. 3). The skull of *Halszkaraptor escuilliei* is only 67.7 mm long, so we do not view its platyrostral skull shape as being strongly indicative of a piscivorous or semi-aquatic lifestyle. Instead, we gravitate towards the hypothesis of [47] that platyrostry represents a movement away from vertebrate carnivory (see following paragraph). To our knowledge, whether a platyrostral snout provides an advantage for semiaquatic life has not been the subject of any study and still requires further investigation.

**Fig. 6:**
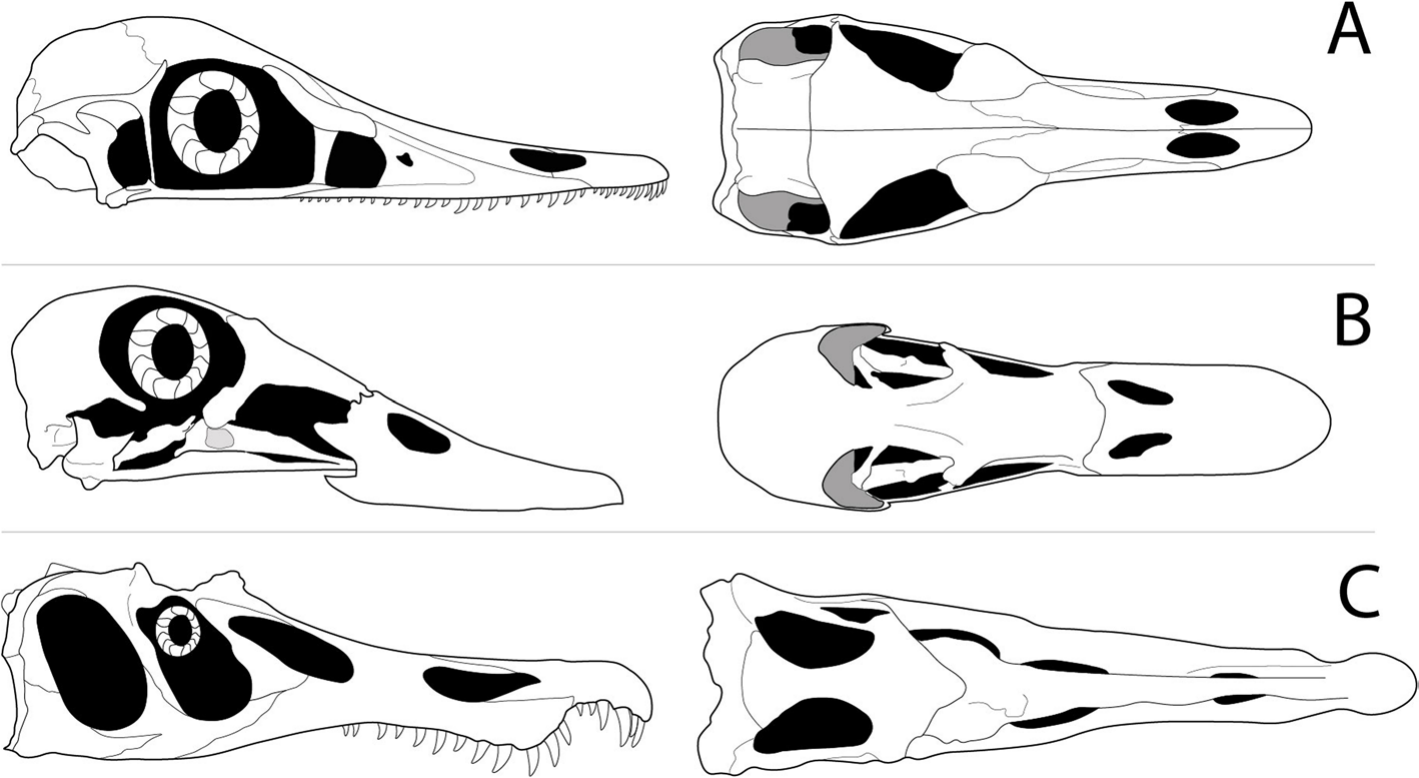
Comparisons of skull shapes between *Halszkaraptor escuilliei*, *Mareca americana*, and *Baryonyx walkeri*. Lateral view (left) and dorsal view (right) of skulls of **A** *Halszkaraptor escuilliei*, **B** *Mareca americana* (American wigeon), and **C** *Baryonyx walkeri*. Line drawing of *Halszkaraptor escuilliei* skull is based on reconstruction of MPC D-102/109 in [10]; *Mareca americana* based on a specimen from Skullsite [34]; and *Baryonyx walker* is based on BMNH R9951 [35]

Extant platyrostral waterfowl, broadly similar in morphology to *Halszkaraptor escuilliei* (Fig. 6), tend to consume primarily invertebrates, fruits, and seeds with a few taxa specialising in leaves [48]. Cau [49] previously suggested the piscivorous sawbill ducks (genus *Mergus*) as analogous to *Halszkaraptor escuilliei*, though we contend these taxa are more longirostrine than platyrostrine. Their rostra are decidedly more mediolaterally narrow than *Halszkaraptor escuilliei* (rostrum width at antorbital fenestra/skull length approximately 0.16 in *Halszkaraptor escuilliei* and 0.05 in *Mergus squamatus*). *Mergus* and its close relatives (Mergini) also represent a relatively recent (< 10 Ma) secondary development of macrocarnivory from herbivorous and filter feeding ancestors [50], further clouding comparisons to a dromaeosaurid whose lineage is generally considered macrocarnivorous. Further investigation is needed into the relationship between platyrostry, feeding ecology, and hydrodynamics. Of particular interest is the pairing of platyrostry with increased neurovascular canals in the premaxilla. Other researchers [46, 47] have pointed out that some theropods with no aquatic affinities have highly-vascularised premaxillae, but to our knowledge no analysis has been done comparing non-avian dinosaurs to multiple extant murky-water-feeding aquatic predators [51–53] that converge upon a highly-vascularized and platyrostral snout. Considering the presence of fluvial deposits rich in silt and mud in the Djadokhta Formation [54, 55], where *Halszkaraptor escuilliei* was discovered, there was murky water present in its paleoenvironment. Therefore, connections between cranial adaptations associated with murky-water-feeding should be further examined as they might provide potential explanations to the unique morphology observed in *Halszkaraptor escuilliei*.

*Halszkaraptor escuilliei* also has a much larger orbit than other dromaeosaurids relative to its skull size (orbit area/total skull area = 0.31, average for Dromaeosauridae = 0.22; see also Fig. 2). The relatively large orbit size could simply be related to the relatively small skull size of *Halszkaraptor escuilliei*; smaller animals tend to have relatively larger eyes [56]. This explanation seems unlikely though given dromaeosaurids with similar skull lengths can have very different relative orbit sizes. For instance, *Tsaagan mangas* and *Velociraptor mongoliensis* have skull lengths of 201 mm and 221 mm and orbit to total skull area ratios of 0.17 and 0.27, respectively. Thus, we suspect the large orbit indicates an ecological adaptation or exaptation. Among archosaurs, circular and relatively large orbits are preserved mainly in smaller or herbivorous species [57]. Orbit length has previously been shown to correlate with eyeball diameter in amniotes, which is a proxy for the size of retina and the number of photoreceptors [58]. In other words, a larger orbit generally indicates an animal with more photoreceptors. On this basis, we propose *Halszkaraptor escuilliei* would have had superior low-light vision to other dromaeosaurids, and may have been more active at lower light conditions (e.g. at night or in murky water). Choiniere et al. [2, 59] previously recovered *Velociraptor mongoliensis* as intermediate between extant diurnal and nocturnal taxa. So *Halszkaraptor escuilliei*, with a larger relative orbit size than *Velociraptor mongoliensis*, would have even greater likelihood of engaging in regular low-light activity. Together with the presence of a highly-vascularized snout [51–53], low-light vision could potentially aid in murky-water-feeding in ephemeral ponds or lakes in *Halszkaraptor escuilliei*’s palaeoenvironment [54].

*Deinonychus antirrhopus* and *Dromaeosaurus albertensis* are the two other frequent outliers in the outlier tests of our shape data (Fig. 3). They both have anteroposteriorly short skulls, snouts, and jugals, as well as dorsoventrally tall snouts. In other words, their skulls are orienirostral. Orienirostral skulls, which are considered the “typical theropod skull shape”, are known to experience lower bending and torsional stresses during anterior bites than playrostral skulls and skulls with more narrow snouts [60, 61]. Hence, one would expect the skulls of *Deinonychus antirrhopus* and *Dromaeosaurus albertensis* to withstand stronger bite forces than dromaeosaurids with either flatter or narrower skulls [60, 61]. Furthermore, anteroposteriorly shorter snouts can reduce the distance between jaw joint and bite point, which leads to the decrease in out-lever distance and increasing the proportion of muscle force transferring to the bitten prey [62–65]. We propose the concave skull roof of *Deinonychus antirrhopus* also contributed to increased bite force by creating room for a larger *m. adductor mandibulae externus profundus* attachment [66]. Snout width, which correlates with the amount of torsional strain a skull would experience [60, 61], also supports these findings. Rostrum width at antorbital fenestra/skull length is 0.231 in *Deinonychus antirrhopus* and 0.254 in *Dromaeosaurus albertensis*, while being at or below 0.16 among other dromaeosaurids with measurable snout width. Although later-diverging dromaeosaurids, such as *Deinonychus antirrhopus*, are believed to have utilised their hindlimbs for prey capture and jaws for subsequent dismemberment [67], having greater bite force could provide additional advantage when it comes to hunting large or struggling prey, increase the speed of prey disassembly and decrease the likelihood of kleptoparasitism, as well as provide advantage when engaging in intraspecific and interspecific competition.

### Allometry of skulls

*Deinonychus antirrhopus* has the largest skull size among all specimens, to the point of being an upper outlier in centroid size (Fig. 4), and *Microraptor zhaoianus* has the smallest. Adult *Deinonychus antirrhopus* are known to have preyed on large terrestrial vertebrates, likely *Tenontosaurus*, based on carbon and oxygen isotope records [68]. An increase in absolute skull size increases gape size and absolute bite force [69–72], both of which would aid in taking large prey. Conversely, one would expect a small skull to limit prey selection. Given that *Microraptor*, which has the smallest skull among our sample, is known to have taken a variety of animal prey [73] and shows macrocarnivorous adaptations [74], we find it unlikely that body size can be used to narrow the ecological interpretations of other dromaeosaurids investigated here. More dietary data of the different dromaeosaurid species will be required in the future to further examine the relationship between skull size and dietary range.

### Mechanical advantage (MA)

The high MA of the temporal muscle group in *Dromaeosaurus albertensis* and *Deinonychus antirrhopus* and the low MA in *Halszkaraptor escuilliei* reflects the shape of their skulls. PGLS results (Table 5) recover PC1 of the shape datasets as significantly correlated with mechanical advantage (MA) generated by the temporal muscle group. An increase in PC1 indicates elongation of the snout, which increases the out-lever length and decreases MA. This spectrum captures the trade-off in potential bite force and jaw closing speed. The decrease in MA in species with positive PC1 score (e.g. *Halszkaraptor escuilliei*) reflects transition to lower bite forces and more quickly-moving jaws. In the same way, the high MA in *Dromaeosaurus albertensis* and *Deinonychus antirrhopus* is explained by their relatively short snout length and enables a relatively stronger bite. Although there has been no published research regarding the prey size of *Dromaeosaurus albertensis*, *Deinonychus antirrhopus* is known to feed on relatively large terrestrial prey [21, 68]. The slower but stronger bites of *Deinonychus antirrhopus* could be beneficial for efficiently and safely dismembering large prey. *Halszkaraptor escuilliei*, on the other hand, would have had a relatively faster jaw-closing speed. Since the skull shape of *Halszkaraptor escuilliei* is more similar to invertivorous waterfowl (see GM discussion), having a fast jaw would have been advantageous for catching smaller elusive invertebrates [75, 76], which is a common food source for waterfowl [48, 77]. The extant platypus *Ornithorhynchus anatinus* is also a platyrostral invertivore [78], and its jaw adductor muscles are also attached more cranially than in other mammals [79] (likely lowering jaw-closing MA), which may indicate this morphofunctional approach has arisen multiple times across amniotes.

PC1 correlates with MA of the temporal muscle group, and most correlations are strong (R^2^_adj_ = 0.749–0.859). This indicates that cranial morphology in an effective predictor of bite performance in these taxa. This is unsurprising, given that PC1 describes the elongation of the skull, which in turn affects the outlever of all MA measurements. Skull size shows no significant correlation with temporal MA in datasets (a) or (c) (*p* > 0.05) for Dromaeosauridae. However, when the outgroup *Gobivenator mongoliensis* is included, skull size shows moderate correlations with temporal MA (R^2^_adj_ = 0.587–0.648, *p* < 0.05) and insignificant correlations with quadrate MA (R^2^_adj_ = − 0.159 – − 0.192, *p* > 0.05) in all of the datasets. More samples are needed to determine if this is simply the result of correlations fluctuating due to a low sample size or differing relationships between size and bite efficiency in Dromaeosauridae and Deinonychosauria overall.

To inspect whether there are associations between the size of fenestrae and MA, we calculated the ratio of cranial fenestrae area to skull model area to test for whether having more open space in the cranium indicates adaptation for weaker bites and thus correlates with lower MA. We did not find any statistically significant result within Dromaeosauridae (R^2^_adj_ = − 0.116 – 0.0516, *p* > 0.3). When individual fenestra, including the orbit, antorbital fenestra, lateral temporal fenestrae, and nares, were tested for correlations with temporal and quadrate muscle group MA, the ratio of orbit area (R^2^_adj_ = 0.635, *p* = 0.0357), antorbital fenestra area (R^2^_adj_ = 0.656, *p* = 0.0315), and lateral temporal fenestrae area (R^2^_adj_ = 0.753, *p* = 0.0157) are all significantly related to temporal muscle group MA (Table S1). For the orbit, the bigger the relative area, the smaller the temporal muscle group MA. A previous study has demonstrated that orbit size is closely related to cranial biomechanics [80]. The smaller the orbit, the more resistant the skull is to bending. This would be beneficial for lowering the strain experienced by the skull while generating strong bite force. Both the antorbital and lateral temporal fenestrae areas show the opposite trend; greater adductor muscle group MA is related to larger fenestra area. For now, we are unable to provide a causal relationship for these trends. The antorbital fenestra is rarely associated with muscle attachment scars [66], and is more generally associated with skull pneumatisation [81]. It has been suggested that the lateral temporal fenestrae could have been a muscle attachment site in non-avian dinosaurs [66], in which case its expansion would be expected to mirror other adaptations to increase bite force, but this hypothesis lacks enough support for us to be confident in this assertion. When the outgroup *Gobivenator mongoliensis* is added to this analysis, the trend for overall cranial fenestrae area and MA stays the same. For individual fenestra, only the size of antorbital fenestra showed significant correlation to MA (R^2^_adj_ = 0.572, *p* = 0.0301). This may indicate the observed relationship between antorbital fenestrae area and MA extends to all Deinonychosauria, a potential avenue for future research.

### Finite element analysis and shape and size of skulls

The three clades of dromaeosaurid modelled for FEA (Velociraptorinae, Dromaeosaurinae, and Halszkaraptorinae) and *Gobivenator mongoliensis* display distinct strain patterns (Fig. 5). Dromaeosaurinae experience greatest tensile strain at the posterior end of the skulls. Relatively high tensile strain are distributed on both the dorsal and ventral edge of the skulls of Velociraptorinae. For “Halszkaraptorinae”, ventral posterior skull experiences the greatest tensile strain. The outgroup *Gobivenator mongoliensis* experiences greatest tensile strain in ventral edge of the skull up to jugal region and posterior ventral end of the skull. Thus, phylogeny appears to be driving the areas in which strain concentrates during a bite. This may be explained by the clade’s different skull shapes. *Halszkaraptor escuilliei* has a distinctive strain distribution in which it experiences low strain along the entire ventral border of the skull. The taxon possesses an elongated and dorsoventrally compressed snout, which is expected to increase the bending and tensile stress of the snout region [61, 82]. *Dromaeosaurus albertensis*, in contrast, has a tall rostrum which resists bending [61, 82], concentrating the reaction force of the bite in the constrained region around the jaw joint. The velociraptorine dromaeosaurids have taller rostra than *Halszkaraptor escuilliei*, so strain is generally restricted to the region cranial to the tooth row. However, these taxa also have larger cranial fenestrae than *Dromaeosaurus albertensis* and thinner borders around these fenestrae. These thinner areas act as strain sinks, reducing the size of the high strain region at the jaw joint and distributing strain throughout the skull. The outgroup *Gobivenator mongoliensis* resembles the velociraptorine strain pattern to an even greater degree, with very large fenestrae causing strain to spread throughout the cranium. This may suggest that the velociraptorine condition is ancestral to Deinonychosauria, though this could also be the result of convergence as the troodontid cranium grew to support larger eyes and brains [43].

However, phylogeny does not explain the quantitative trends in strain, as there is no significant phylogenetic signal in the dromaeosaurid intervals data (K_mult_ = 0.543, *p* = 0.423). Trends in the percent area of dromaeosaurid skulls under a given level of strain (Fig. 5H) also do not correlate with phylogeny. *Dromaeosaurus albertensis*, *Halszkaraptor escuilliei*, and *Velociraptor mongoliensis* represent three different dromaeosaurid families, but their strain distributions are more similar than *Velociraptor mongoliensis*’ to any other velociraptorines. In the same vein, the distinct descending strain pattern of *Deinonychus antirrhopus* is shared only with *Gobivenator mongoliensis*, which is not a dromaeosaurid. In other words, while phylogeny appears to determine which anatomical regions of the dromaeosaurid skull act as strain sinks, it does not determine their adaptations for bite resistance overall nor the relative area under given levels of strain.

Quantitative strain analysis also produces paradoxical results given other lines of ecological evidence. As noted above (GM Discussion), *Deinonychus antirrhopus* is assumed to be macrocarnivorous from a variety of evidence [21, 68]. And yet, *Deinonychus antirrhopus* experiences the highest MWAM strain (579 με) of any dromaeosaurid examined here. This indicates a relatively low resistance to bite force in the skull, unexpected for an animal pursuing large and struggling prey. Conversely, *Halszkaraptor escuilliei* that likely fed on much smaller prey based on evidence presented here and in [10, 49], experiences the second lowest MWAM strain (386 με) of any dromaeosaurid modelled and thus the highest resistance to bite forces. The latter case, at least, has some explanation. *Halszkaraptor escuilliei*, in broad terms, parallels the morphological trajectory of hummingbirds in that its rostrum is elongated and its jaw adductor origins are shifted cranially relative to similar taxa [83]. As such, as [83] noted, the finite element model experiences lower strains because the animal’s biting muscles are transferring relatively little force into the jaw and, in turn, bitten objects. This is consistent with reconstruction of *Halszkaraptor escuilliei* as hunting small and easy-to-process invertebrate prey. The explanation for high strain in *Deinonychus antirrhopus* is less clear. It could be that, like many extant varanoid lizards [84, 85], *Deinonychus antirrhopus* did not use bite force to disassemble prey but rather relied on neck-driven pullback disassembly. Some though, like [86], have suggested varanoids could only develop this feeding style with the aid of venom which is currently not known in any dinosaur [87]. The combination of high MA and high MWAM strain could indicate adaptations for flexibility in the skull itself. In reconstructions of *Deinonychus antirrhopus* as an active hunter, this could aid in increasing effective gape as prey is disassembled, in turn increasing the speed at which prey is disassembled and minimising risks of kleptoparasitism. However, if these adaptations are for either pullback disassembly or increasing effective gape, this raises the question of why other dromaeosaurids would not share these adaptations. We suggest different levels of scavenging between species may be an influence. *Velociraptor mongoliensis* experiences the lowest MWAM strain (360 με) of any modelled dromaeosaurid, and this taxon is suggested to have had regularly engaged in scavenging behaviour [88]. Higher jaw strength is associated with scavenging among extant carnivorous birds [83, 89] and mammals [90, 91], so it may be that dromaeosaurids with stronger jaws (e.g. *Dromaeosaurus albertensis*, 399 με) were more reliant on carrion and those with weaker jaws (e.g. *Tsaagan mangas*, 550 με) were more reliant on freshly-killed prey. Additional lines of evidence like endocranial mapping [88] could help to evaluate this hypothesis in the future.

A previous study [82] has suggested that skull length is positively related to relative stress experienced by theropod crania during a bite. We performed a regression comparing skull length to MWAM strain and log skull length to log MWAM strain (Fig. S2) to see if this trend persists in dromaeosaurids. There is no statistically significant relationship between MWAM strain and skull length (R^2^_adj_ = 0.099, *p* = 0.281) or log MWAM strain and log skull length (R^2^_adj_ = 0.0106, *p* = 0.363) in dromaeosaurids. The low statistical power of our small sample size could be the cause of the statistical insignificance, though even subjectively the data spread appears random (Fig. S2). Thus, our findings do not conform to those of Rayfield [82]. It bears mentioning that the taxa in Rayfield [82] are larger (~ 500–1700 mm long) than the dromaeosaurids studied here (67.7–303 mm long). Miller and Pittman [92] proposed that stress and strain may be less important in smaller animals, as work scales with mass at a higher rate than fracture surface area. Additionally, we note that smaller skulls are more able to eat around hard parts of carcasses and so face less fracture risk from interacting with prey bones or integument. Thus, size may explain the difference in results between our data and those of Rayfield [82]. If fracture is less of a risk in dromaeosaurid-sized animals, then those with similar skull lengths are less mechanically constrained in their skull architecture than larger theropods. This means they can experience a larger range in relative strain during biting. This is further supported by the continued lack of significant correlation between log skull length and MWAM strain when the outgroup, whose skull is 183 mm long, is included (R^2^_adj_ = − 0.0591, *p* = 0.452).

We note briefly that future research should aim to include the pterygoideus muscle group in biomechanical studies of the jaw. The pterygoideus muscle group assists in jaw closing in theropods during feeding, which affects bite force generation and regulates palatal movement [66, 93]. Although insertions located on the lower mandible could be located in specimens used in this study with complete articular, angular, and surangular bones, origin and insertion sites locate on the pterygoid bone are difficult to determine without ventral views of the upper cranium [66]. *M. pterygoideus dorsalis* also originates from the lateral surface of the ectopterygoid [66], which is not visible in many of the specimens included in the current study. For the many species known from compression fossils, the pterygoid and ectopterygoid are completely unknown. While we believe our models incorporating the temporal and quadrate muscle groups provide a useful exploration of dromaeosaurid cranial biomechanics, inclusion of the pterygoideus muscle groups will allow future works to create an even more complete picture of the bite mechanics of dromaeosaurids, troodontids and other theropods.

## Conclusions

Dromaeosaurids have traditionally been depicted as morphologically and ecologically conservative theropods [3, 4], and our findings do not wholly contradict this. The axes of morphological disparity we recovered in their skulls are more restricted than is typical for non-avialan theropods, and FEA intervals data show relatively little variation in the distribution of strain during modelled biting. There appears, then, to be developmental constraints on dromaeosaurid skulls that keep them within a continuum of form and function. Within this continuum, though, there is still variety beyond the classic idea of dromaeosaurids as active hunting macropredators. At one end of this continuum is *Deinonychus antirrhopus*, fitting the traditional depiction of a ‘raptor’ with a skull that is tall and short and with several adaptations for strong bite forces and established evidence of macrocarnivory. On the other end is *Halszkaraptor escuilliei* with a flat and long skull, whose ecology is less understood. We do not support past proposals of piscivory in this taxon. Instead, we suggest a lower trophic level based on comparisons with extant platyrostral waterfowl, but cannot support or refute a general semi-aquatic habit. We highlight adaptations of *Halszkaraptor escuilliei* for low-light conditions and suggest this may indicate activity at night or in murky water. Within the dromaeosaurid morphofunctional gradient, there may be a corresponding ecological gradient as suggested by its two endmembers, or multiple ecological niches may have been available at any given point within the morphofunctional gradient. Bite performance appears only weakly related to a taxon’s position along the morphological gradient, and aspects such as methods of prey disassembly or level of carrion consumption may instead drive load resistances. We suspect the region of this continuum occupied by *Halszkaraptor escuilliei* and *Velociraptor mongoliensis* approximates the ancestral skull shape, given their general similarity to the troodontid outgroup *Gobivenator mongoliensis*, with taller-snouted dromaeosaurids like *Deinonychus antirrhopus* representing a derived condition.

Despite considering hundreds of specimens for our study, we were only able to analyse ten dromaeosaurid specimens with sufficiently complete cranial material. The limitations this imposed on the statistical power of our analyses means that several factors in shape and function could not be reliably investigated, including phylogenetic signal [3, 4] and morphological integration and modularity [38, 39]. Thus, further discovery and publication of more complete dromaeosaurid cranial material, especially from Dromaeosaurinae and “Halszkaraptorinae”, is essential to generate a more complete picture of the relationship between dromaeosaurid skull shape, skull mechanics, and ecology. We suspect that future work with expanded datasets will find that dromaeosaurid skull shapes reflect a mix of shared evolutionary history and functional adaptations, and that our proposed sequence of cranial evolution will be refined as the dromaeosaurid fossil record is expanded. Additional understanding of the subtleties of dromaeosaurid ecology, for instance through isotope analyses [94] or expansion of feeding trace studies [21], will also aid in calibrating the morphofunctional data to corresponding ecologies. Despite these caveats, this study demonstrates the ability of skull shape and functional metrics to discern non-avialan theropod ecology at lower taxonomic levels and identify variants of carnivorous feeding in the fossil record. This study framework may therefore be of interest in the study of other theropod groups and potentially more distantly related vertebrates as well.

## Methods

### Sampling

Among the 52 recognised dromaeosaurid species [16, 24, 25, 27, 95–98], 32 species are solely represented by postcranial skeletons with the remaining 20 represented by both cranial and postcranial skeletons. Among the 20 species with cranial material, nine species are represented by skulls complete enough to perform morphological and functional analyses. As a result, nine species (10 specimens) representing three of the four subfamilies within Dromaeosauridae were included in the analyses (Table 1), with Unenlagiinae being the only subfamily unrepresented. Only adult specimens were used.

### Geometric morphometrics

We applied two-dimensional geometric morphometrics (GM) to quantify the differences in cranial shape of the selected specimens (Fig. 1). Landmarks and semi-landmarks were assigned to capture overall shape of the skull and fenestrae rather than only capturing shape variations of specific morphological characters which we hypothesised to be ecologically informative a priori. With that in mind, we have also produced a table of biological variables which each landmark could potentially help to characterise (Table S5). For most specimens, we used photos or CT scan images of the published specimens for the landmarking procedure (Table 1). All photographs were inspected for signs of image warping and to ensure they were in lateral view. One of us (MP) has seen most specimens in-person and confirmed the images accurately reflect their anatomy. We expect all utilised images to be comparable to one another. We took into consideration that some of the poorly preserved specimens are fragmentary. Hence, if reconstructed skull casts are available, they were used for landmarking instead of the fragmented original specimens (including casts of *Deinonychus* and *Dromaeosaurus*). We initially selected 13 Type I and 11 Type II homologous traditional landmarks and a total of 231 semi-landmarks to capture the overall shape of the lateral view of the skulls, including shape of orbit and antorbital fenestrae, based on previous publications on GM of theropod skulls [3, 4]. We selected 12 traditional landmarks from [3] and added 12 new traditional landmarks for better capturing Dromaeosaurid cranium (Fig. 1). However, the poor preservation of some of the cranial materials limits the total number of landmarks that could be included. Thus, there is a trade-off between the number of species included in the analysis and the resolution of shape data obtained from these species. To maximize the data captured, we collected three sets of landmarks data: (a) 11 specimens representing ten species using 52 landmarks (10 traditional landmarks; six curves of seven semi-landmarks), (b) ten specimens representing nine species using 76 landmarks (13 traditional landmarks; nine curves of seven semi-landmarks), and (c) seven specimens representing seven species using 206 landmarks (19 traditional landmarks; 17 curves of 11 semi-landmarks). Some of the published specimens do not have reconstructed casts available but rather interpretative illustrations of the reconstructed skulls. Thus, in addition to the three datasets from above, we included a fourth dataset (d) which is based on a combination of published fossil specimens and illustrations of the reconstructions of skulls. If published reconstruction illustrations were available for skulls without well-preserved original specimens nor reconstructed casts, illustrations would be used to represent the specimen for landmarking procedure. If reconstruction illustrations could allow the inclusion of a greater number of landmarks, they were also used to represent the specimens rather than images of the original specimens. Dataset (d) includes eight specimens representing eight species using 230 landmarks (21 traditional landmarks; 19 curves of 11 semi-landmarks). Since the reconstruction of *Sinornithosaurus millenii* by Xu and Wu [99] was based on a highly fragmented fossil, and this reconstruction contradicts the morphology seen in more complete specimens discovered since [24], we consider this reconstruction dubious. For completeness, Table S6 provides results of analyses with this reconstruction added to dataset d. For ease of communication, the datasets above are labelled as dataset with least number of landmarks (a), intermediate number of landmarks (b), largest number of landmarks (c), and the reconstruction dataset (d) (Table 2).

Sensitivity tests were performed by subsampling individual curves of semi-landmarks in order to reduce noise while accurately representing the shape of the skeletal materials of the skull in a relative warps plot. We used tpsUtil v.1.58 to create TPS files of all the specimens [100]. We then digitized landmarks and semi-landmarks using tpsDig 2 v.2.16 [101]. All landmarks are size-calibrated using the scale included in individual specimen photos and reconstruction drawings.

### Analyses of gm data

#### Principal component analysis

We analysed the GM data in R 4.0.2 [102] using the packages geomorph 4.0.4 [103], lmodel2 1.7–3 [104], MASS 7.3–53 [105], Morpho 2.8 [106], multcomp 1.4–15 [107], shapes, 1.2.6 [108], and vegan 2.6–2 [109]. We applied generalised Procrustes superimposition [110, 111] to translate, scale, and digitize the landmarks of all specimens. We then analysed the data using principal component analyses (PCA) to inspect the morphospace occupied by different species. Our small sample sizes limit the statistical power of any parametric statistical analyses, such as t-test and ANOVA. So to estimate group means and identify individual species that deviate from the norm in cranial shape, we created boxplots for all PC scores from each of the four datasets as non-parametric outlier tests [112, 113]. We created the boxplots using the inbuilt R graphics package (4.0.2) [102].

#### Centroid size analysis

We quantified the size of the skulls using centroid size and performed outlier tests with boxplots for all four datasets to identify species that are anomalously large or small (1.5 times the interquartile range below the first quartile or above the quartile range). Skull shapes are often correlated with size, with size and skull shape known to covary from previous studies on birds and mammals [114, 115]. Moreover, larger species of both reptiles [71, 116] and mammals [65, 69] are known to produce greater bite force, which should expand the range of dietary possibilities [117] and may aid during intra- and interspecific competition [118]. Thus, differences in skull size are expected to be ecologically important.

### Mechanical advantage

For each of the skulls and skull reconstructions in Table 1, we calculated anterior jaw-closing mechanical advantage (MA) as a proxy for bite force and speed [20]. MA is calculated as the in-lever divided by the out-lever. Distance of in-lever was measured from jaw joint to insertion of muscles on the lower jaw, and out-lever was measured from jaw joint to a bite point at the rostralmost tooth [119]. Posterior jaw-closing MA was briefly investigated but ultimately excluded due to uncertainty in the location of the cranialmost tooth in some taxa. In dromaeosaurids which we were able to measure posterior jaw-closing MA, it correlated strongly with anterior jaw-closing MA (R^2^ = 0.78 for temporal group and R^2^ = 0.76 for quadrate group). Thus, we assume that the anterior measure accurately represents relative bite force adaptations throughout the jaw.

In theropods, cranial muscles responsible for generating bite force can be separated into three groups: (1) the temporal group consisting of *m*. *adductor mandibulae externus superficialis* (mAMES), *m*. *adductor mandibulae externus medialis* (mAMEM), *m*. *adductor mandibulae externus profundus* (mAMEP), and *m. pseudotemporalis superficialis* (mPSTs); (2) the quadrate group which consists of *m*. *adductor mandibulae posterior* (mAMP) and *m. pseudotemporalis profundus* (mPSTp); and (3) the pterygoid group consisting of *m. pterygoideus* (mPT) [66, 72, 120]. MA was calculated separately for insertions of the temporal and quadrate groups to investigate possible differences in biting style; pterygoid group measurement points are not visible in lateral view so MA for this group is not measured. MA is compared both directly between taxa and with skull shape via phylogenetic generalized least squares (PGLS; see below).

### Finite element analysis

We created two-dimensional finite element models of the lateral view of six dromaeosaurid upper jaws based on images of published specimens, listed in Table 1. We solved our models using Optistruct in Hypermesh (2022 version) [121]. As bone thickness is required to accurately use planar stress assumptions [92, 122], which is not available for the specimens included in our study, we used planar strain assumptions which require no thickness assumptions [123]. This assumption set posits that strain is negligible mediolaterally in the body, which we believe is reasonable for the initial bite of a dromaeosaurid. For material properties of the skulls, we used a Young’s modulus of 20.49 GPa and a Poisson’s ratio of 0.4 following previous FEA of non-avialan theropods [1, 124].

During a bite, all muscles should exert force at roughly the same time. Hence, we set up a single load condition including both the temporal muscle group and quadrate muscle group as model loads (Fig. 2). We were not able to include the pterygoid muscle group into our models due to preservation obscuring attachment sites for *m. pterygoideus*. Since small theropods likely rely more on anterior bite to create slashing bite for killing small prey [119], all models were constrained at the most anterior tooth in dorsoventral movement and at the jaw joint in all directions of movement to simulate an anterior bite.

To restrict the analysis to showing only the functional effects of shape variation of the skulls (rather than size differences), the applied loads of all models were scaled based on their area. We applied equation from Table 2 in Marcé-Nogué et al. [122] for scaling forces in planar strain models to achieve a constant stress state:

$${F}_B={F}_A\ast \sqrt{\left({A}_B/{A}_A\right)}$$

where A_A_ = area of reference model, A_B_ = area of the new model, F_A_ = force applied to the reference model, F_B_ = force applied to the new model. We selected *Halszkaraptor escuilliei* as the model to use as A_A_ because it is the smallest specimen among the six dromaeosaurid species studied. The arbitrary force (F_A_) applied to the reference model was 30 N based on previous published bite force estimations of dromaeosaurid theropods [120], Aves [125, 126], and mammals [127] of similar sizes.

We obtained strain values for all of the elements in each model. To take into account the size differences of the elements within and between the models, we calculated the mesh-weighted arithmetic mean (MWAM) strain for each model [128]. We also applied the intervals method [129] to quantitatively compare the distribution of strain magnitudes in our models. Our sample size was too small to produce informative multivariate graphs, so instead we compared intervals data from our models with a bar plot as in [130]. In addition, the area of the fenestrae in each model were recorded using Hypermesh. Each of these were compared directly between taxa and to skull shape via PGLS (see below).

### Phylogenetic comparative methods

To take into account the shared evolutionary history of the closely related taxa, we employed phylogenetic comparative methods to investigate the correlations between phylogeny and the shape and functional data collected. We calculated K_mult_ to estimate the phylogenetic signal present in our shape, MA, and FEA intervals dataset [131]. A K_mult_ larger than one indicates that closely related species are more similar to each other than expected under the Brownian motion model of evolution. A K_mult_ smaller than one indicates closely related species resemble each other less than expected under the Brownian motion model. In order to identify the strength of phylogenetic signals of the PCs, we have also calculated Blomberg’s K for individual variables from all datasets [132]. Blomberg’s K is interpreted in the same way as K_mult_.

In addition, we compared MA results, and FEA results to centroid size and our shape data via phylogenetic generalized least squares (PGLS) to determine how shape and size affect these metrics. PGLS allows us to investigate possible correlations between these metrics within a framework of phylogenetic non-independence [133]. For all regressions, all PCs of the GM data were used as the independent variable. For MA, MA of each muscle group was regressed individually against shape and centroid size data. For FEA, MWAM strain data was regressed against shape and centroid size. All morphological characters were assumed to evolve under Brownian motion.

We calculated K_mult_ using the R package geomorph 4.0.4 [103], calculated Blomberg’s K using picante 1.8.2 [134], and performed PGLS using caper 1.0.3. Note that the R^2^_adj_ values reported for PGLS are a pseudo- R^2^_adj_ which approximates the R^2^_adj_ value of normal linear regression [135]. We constructed the dromaeosaurid phylogeny based on Ding et al. [42], in which the authors have provided both tree topology and branch length of all but one species included in this study. The only species whose position could not be obtained directly from Ding et al. [42] was *Halszkaraptor escuilliei*. We estimated its age range was using the occurrence data in the Paleobiology Database [10, 136]. This phylogeny was constructed and applied to the two phylogenetic comparative methods using phytools 1.0–3 [137] and ape 5.6–2 [138].

### Supplementary Information

**Supplementary Material 1.**

**Supplementary Material 2.**

## Data Availability

All processed data supporting the findings of this study are available within the paper and its Supplementary Information. Raw measurements and R code used to process the data are in preparation for submission by the time of publication to a repository. These data are available to reviewers upon request.
