# Supplementary material for: Morphological disparity and structural performance of the dromaeosaurid skull informs ecology and evolutionary history
Source: BMC Ecol Evol. 2024 Apr 16;24:39. doi: 10.1186/s12862-024-02222-5 (PMC11020771; doi:10.1186/s12862-024-02222-5)
Supplement: Supplementary file 1 — Supplementary Material 1. [file 12862_2024_2222_MOESM1_ESM.docx]

**Supplementary figures and table**

Tables S1 through S4 can be found in a separate Excel document.

**Table S5. Landmarks (L) and semi-landmarks (SL) of 2D geometric morphometric analysis and the biological variables they characterize**

**Figure S1. All significant outlier PCs based on outlier analyses of each of the datasets**

- PC6, PC8 of dataset (a)
- PC4, PC9 of dataset (b)
- PC6 of dataset (c)
- PC6, PC7 of dataset (d)

**Figure S2. Skull length to MWAM strain and Log skull length to MWAM strain regression graph**

**Figure S3. PCA figures showing trajectory observed**

- PC1 and PC2 of dataset (c) and (d) (with outgroup)
- PC1 and PC2 of dataset (c) and (d) (without outgroup)

**Figure S4. PCA graphs of each dataset after excluding landmarks on top of the cranium**

**Table S5. Landmarks (L) 2D geometric morphometric analysis and the biological variable they characterize**

| **Landmarks and semi-landmarks** | **Biological variable characterized** |
| --- | --- |
| L1. Anterior end of premaxilla | Total skull length |
| L2. Junction of premaxilla and maxilla | Length of premaxilla |
| L3. Ventral end of rostrum corresponding to anterior end of maxillary fenestra | Overall shape and curvature of the skull |
| L4. Ventral end of rostrum corresponding to anterior end of antorbital fenestra | Overall shape and curvature of the skull |
| L5. Junction of maxilla and jugal | Length of maxilla |
| L6. Anterior end of antorbital fenestra | Shape of antorbital fenestra |
| L7. Posterior dorsal end of antorbital fenestra | Shape of antorbital fenestra |
| L8. Posterior ventral end of antorbital fenestra | Shape of antorbital fenestra |
| L9. Anterior end of orbit | Shape of orbit |
| L10. Junction between postorbital and orbit | Shape of orbit |
| L11. Dorsal most point of orbit | Shape of orbit |
| L12. Junction between jugal and quadratojugal | Length of jugal |
| L13. Junction between quadratojugal and quadrate | Length of quadratojugal |
| L14. Posterior ventral end of quadrate | Length of ventral end of skull |
| L15. Posterior dorsal end of quadrate | Height of quadrate |
| L16. Posterior end of squamosal | Length of squamosal protrudes out posteriorly |
| L17. Posterior dorsal end of parietal | Height of posterior end of skull and length of dorsal end of skull |
| L18. Junction of frontal and parietal | Length of parietal |
| L19. Junction of nasal and frontal | Length of frontal |
| L20. Dorsal end of rostrum corresponding to anterior end of antorbital fenestra | Overall shape and curvature of the skull |
| L21. Dorsal end of rostrum corresponding to anterior end of maxillary fenestra | Overall shape and curvature of the skull |

***Aim of the semi-landmarks assigned is to capture overall shape of the skull and shape of the fenestrae that are identifiable in the majority of the specimens

***There is no semi-landmarks between L13 to L14. This is because L13 and L14 overlap with each other in some of the specimens, which makes it impossible to assign semi-landmarks

***There is no semi-landmarks between L21 to L1 because some of the specimens have a portion of the nasal missing or displaced

**Figure S1. All significant outlier PCs based on outlier analyses of each of the datasets**

PC6 of dataset (a) with deformation grids of positive and negative PC6. 1: Deinonychus, 2: Dromaeosaurus, 3: Halszkaraptor, 4: Linheraptor, 5: Microraptor, 6: Saurornitholestes, 7: Tsaagan, 8: Velociraptor a, 9: Velociraptor b, 10: Sinornithosaurus, 11: Gobivenator

Negative PC6 Positive PC6

PC8 of dataset (a) with deformation grids of positive and negative PC8. 1: Deinonychus, 2: Dromaeosaurus, 3: Halszkaraptor, 4: Linheraptor, 5: Microraptor, 6: Saurornitholestes, 7: Tsaagan, 8: Velociraptor a, 9: Velociraptor b, 10: Sinornithosaurus, 11: Gobivenator

Negative PC8 Positive PC8

PC4 and PC5 of dataset (b) with deformation grids of positive and negative PC4. 1: Deinonychus, 2: Dromaeosaurus, 3: Halszkaraptor, 4: Linheraptor, 5: Microraptor, 6: Saurornitholestes, 7: Velociraptor a, 8: Velociraptor b, 9: Tsaagan, 10: Gobivenator

Negative PC4 Positive PC4

PC8 and PC9 of dataset (b) with deformation grids of positive and negative PC9. 1: Deinonychus, 2: Dromaeosaurus, 3: Halszkaraptor, 4: Linheraptor, 5: Microraptor, 6: Saurornitholestes, 7: Velociraptor a, 8: Velociraptor b, 9: Tsaagan, 10: Gobivenator

Positive PC9 Negative PC9

PC5 and PC6 of dataset (c) with deformation grids of positive and negative PC6. 1: Deinonychus, 2: Dromaeosaurus, 3: Halszkaraptor, 4: Linheraptor, 5: Tsaagan, 6: Velociraptor, 7: Gobivenator

Negative PC6 Positive PC6

PC6 and PC7 of dataset (d) with deformation grids of positive and negative PC6 and PC7. 1: Deinonychus, 2: Dromaeosaurus, 3: Halszkaraptor, 4: Linheraptor, 4: Saurornitholestes, 6: Tsaagan, 7: Velociraptor, 8: Gobivenator

Negative PC6 Positive PC6

Negative PC7 Positive PC7

**Figure S2. Skull length to MWAM strain and Log skull length to MWAM strain linear regression graph**

Linear regression between skull length (mm) and MWAM strain for specimens with MWAM strain values available. (R^2^_adj_ = -0.0844, p = 0.498)

Linear regression between log skull length (mm) and log MWAM strain for specimens with MWAM strain values available. (R^2^_adj_ = -0.0591, p = 0.452)

**Figure S3. PCA figures showing trajectory observed**

PC1 and PC2 of dataset (c) including outgroup *Gobivenator*

PC1 and PC2 of dataset (d) including outgroup *Gobivenator*


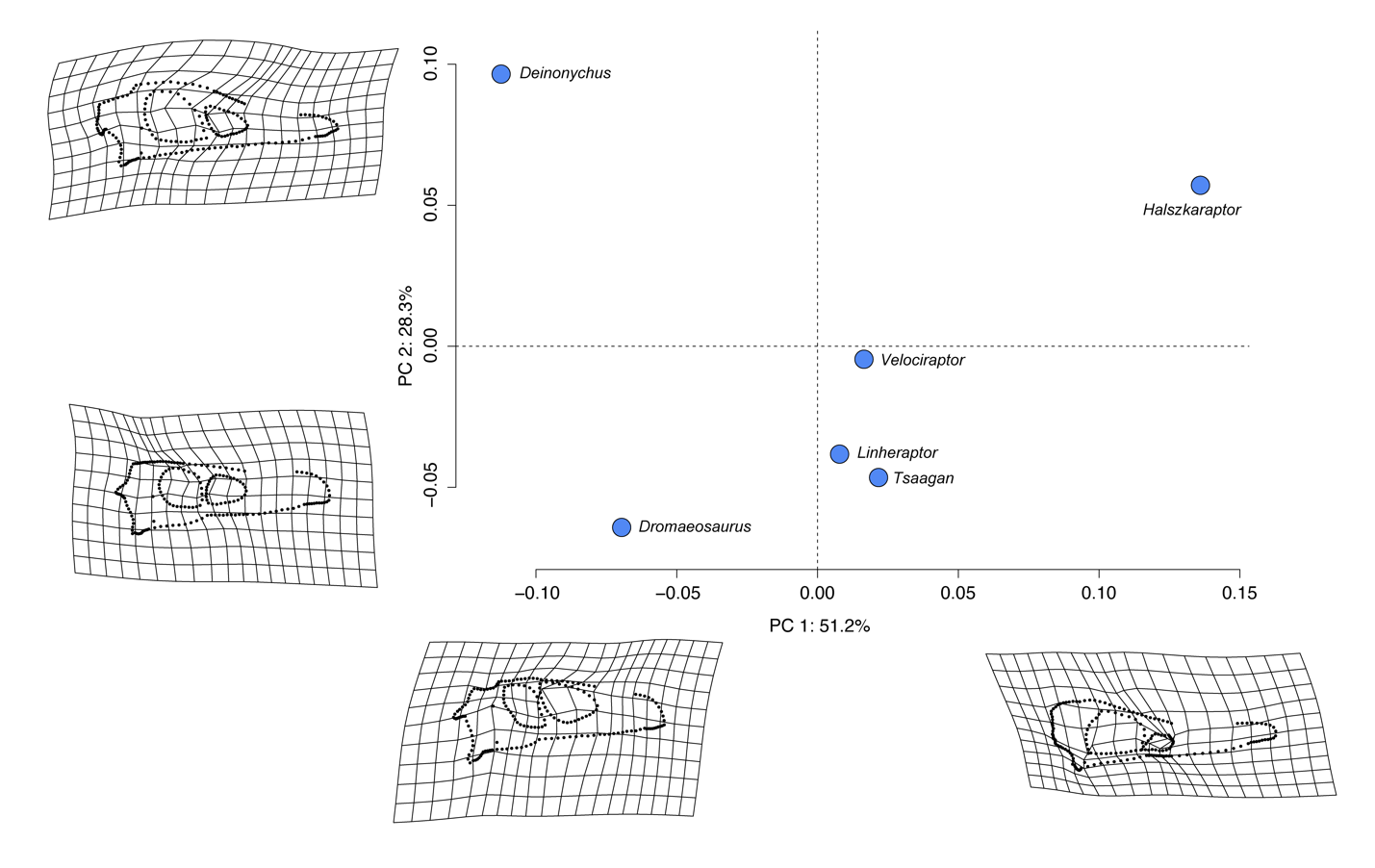


PC1 and PC2 of dataset (c) excluding outgroup *Gobivenator*

PC1 and PC2 of dataset (d) excluding outgroup *Gobivenator*

**Figure S4. PCA graphs of each dataset after excluding landmarks on top of the cranium**

Dataset (a)

1: Deinonychus, 2: Dromaeosaurus, 3: Halszkaraptor, 4: Linheraptor, 5: Microraptor, 6: Saurornitholestes, 7: Tsaagan, 8: Velociraptor a, 9: Velociraptor b, 10: Sinornithosaurus, 11: Gobivenator

Dataset (b)

1: Deinonychus, 2: Dromaeosaurus, 3: Halszkaraptor, 4: Linheraptor, 5: Microraptor, 6: Saurornitholestes, 7: Velociraptor a, 8: Velociraptor b, 9: Tsaagan, 10: Gobivenator

Dataset (c)

1: Deinonychus, 2: Dromaeosaurus, 3: Halszkaraptor, 4: Linheraptor, 5: Tsaagan, 6: Velociraptor, 7: Gobivenator

Dataset (d)

1: Deinonychus, 2: Dromaeosaurus, 3: Halszkaraptor, 4: Linheraptor, 4: Saurornitholestes, 6: Tsaagan, 7: Velociraptor, 8: Gobivenator

This dataset uses 255 landmarks examining 8 species. Together, PC1 and PC2 explain 64.81% of the cranial morphological variations observed among the eight specimens (PC1: 41.7%; PC2: 23.1%) (Fig. 9). Positive PC1 scores describe an anteroposteriorly elongate skull, snout, and jugal; a dorsoventrally shorter posterior end of skull; a rounded dorsal and posterior end of the skull; anteroposteriorly and dorsoventrally short antorbital fenestra; and a dorsoventrally less expanded infratemporal fenestra, and a slightly dorsoventrally compressed quadrate curving towards posterior end of the skull. Positive PC2 scores describe a dorsoventrally compressed snout, a flat ventral edge of the skull, an anteroposteriorly expanded and concave squamosal-parietal region, a dorsoventrally curved quadrate, a slightly triangular antorbital fenestrae, and a slightly rectangular orbit.

In outlier tests, PC4, PC6, and PC7 each have one outlier identified (Fig. 10). For PC4, *Saurornitholestes langstoni* represents the outlier with negative PC is lower outlier. Positive PC4 scores describe a curved jugal and nasal, a posteriorly-protruding squamosal, and a less posteriorly expanded orbit (Fig. S6). For PC6, *T. mangas* is an upper outlier (Fig. S7). Positive PC6 scores describe a dorsoventrally flat jugal and frontal, a dorsoventrally short and anteroposteriorly elongate orbit, and an enlarged antorbital fenestra. For PC7, *V. mongoliensis* is an upper outlier. Positive PC7 scores describe a rounded parietal, reduced lateral temporal fenestra, an anteriorly-expanded orbit, a dorsoventrally-compressed infratemporal fenestra, and a posteriorly-protruding squamosal (Fig. S8). Although no outliers have been identified for PC1 and PC2, the standard deviation for both are visibly larger than other PCs (Fig. 10).
